# Consumer perceptions of strain differences in *Cannabis* aroma

**DOI:** 10.1371/journal.pone.0192247

**Published:** 2018-02-05

**Authors:** Avery N. Gilbert, Joseph A. DiVerdi

**Affiliations:** 1 Headspace Sensory, LLC, Fort Collins, Colorado, United States of America; 2 XTR Systems, LLC, Fort Collins, Colorado, United States of America; Barnard College, UNITED STATES

## Abstract

The smell of marijuana (*Cannabis sativa* L.) is of interest to users, growers, plant breeders, law enforcement and, increasingly, to state-licensed retail businesses. The numerous varieties and strains of *Cannabis* produce strikingly different scents but to date there have been few, if any, attempts to quantify these olfactory profiles directly. Using standard sensory evaluation techniques with untrained consumers we have validated a preliminary olfactory lexicon for dried cannabis flower, and characterized the aroma profile of eleven strains sold in the legal recreational market in Colorado. We show that consumers perceive differences among strains, that the strains form distinct clusters based on odor similarity, and that strain aroma profiles are linked to perceptions of potency, price, and smoking interest.

## Introduction

*Cannabis sativa* L. has been cultivated for millennia as a source of fiber and edible seeds, and also for the psychotropic effects of Δ^9^-tetrahydrocannabinol (THC) [1]. Today there exist many varieties and strains bred for desirable traits such as potency, growth form, and flavor. Although cannabis remains on Schedule I of the Controlled Substances Act in the United States, a number of individual states have legalized its possession for medical and recreational use. As a result, a thriving retail industry has emerged. Among other marijuana-based products, dispensaries sell dried cannabis flowers or “buds” that, in addition to varying in THC content, exhibit a wide range of strain-specific aromas.

The smell of marijuana is an important issue on many fronts, from the “in plain smell” rule adopted as a Fourth Amendment search-and-seizure doctrine in many jurisdictions [2] to public nuisance complaints arising from commercial growing operations [3]. With the expansion of legal recreational use, producers and consumers are becoming more aware of the nuances of cannabis aroma for aesthetic and experiential reasons, as well as for cues it may provide to product quality. For example, aroma and flavor are among the factors judged in organized cannabis competitions [4]. Given the presumed influence of microclimate and soil conditions on cannabis quality, efforts are underway in California to establish appellations similar to those for wine; eleven cannabis appellations have been proposed for Mendocino County, and Humboldt County is pursuing a similar plan [5, 6].

Validated sensory lexicons are available in product categories such as wine [7], coffee [8], and beer [9]. These lexicons, often presented visually in the form of “flavor wheels,” provide standardized sets of terms that enable concise, reliable and easily understood descriptions of a product’s chief sensory attributes. Empirically-based lexicons are useful to experts and novices alike, and help consumers appreciate the many versions of the finished product. No such lexicon exists for the aroma of dried cannabis flower nor, to our knowledge, has the topic been examined from a primarily psychophysical perspective. Scientific analysis of the scent has been confined almost exclusively to chemical identification of the volatile compounds found throughout the plant but notably in the resin of the female inflorescence [10]. Chemical analysis provides useful information but, by itself, offers little insight as to how a given sample smells. At some point, sensory judgments become necessary to link chemical composition to olfactory character.

Here we undertake direct sensory evaluation as a first step to an empirical understanding of cannabis aroma. Our goals were to: (a) develop an initial olfactory lexicon for cannabis bud using standard sensory research methods; (b) determine whether there are measurable differences in aroma profile among strains available on the retail market; and (c) determine whether strain-specific aroma profiles can be linked to consumer perceptions of product quality.

## Materials and methods

### Ethics statement

This study was approved by the Western Institutional Review Board (Puyallup, WA) (WIRB Protocol #20170080). All participants provided informed written consent using a form approved by WIRB. At no time did participants come into direct contact with the cannabis samples. Retail sale of marijuana for recreational use to adults 21 years of age and older has been legal in the state of Colorado since January 1, 2014.

### Participants

Test participants were recruited from the local community by means of fliers and an online newspaper advertisement. Printed text emphasized “current, former, and non-users all welcome” and that only sniffing was required (“no touching, no smoking, no eating”). All participants were at least 21 years of age, residents of Colorado, and had a self-reported normal sense of smell. Exclusion criteria included self-reported pregnancy, active nasal allergy, and current head cold. Subjects were paid $20.00 for their participation.

### Odor stimuli

Cannabis samples in the form of dried flower were purchased from two Fort Collins dispensaries licensed for retail recreational sale by the state of Colorado: Solace Meds and Infinite Wellness Center. Eleven strains were purchased: Alien Dawg, Durban Poison, Fruity Pebbles, G13, Jilly Bean, Lamb’s Breath, Lemon Diesel, Mob Boss, OG Kush, Snoop OG, and Super Skunk. (Details regarding the dispensaries, cultivation facilities, and on-label information about the strains are provided in S1 Note. Source and specification of study materials.) These strains were selected to cover a wide range of odor character, based on information from web-based “strain reviews” as well as pre-purchase sniff sampling at the dispensaries by the senior author. One strain (Durban Poison) was purchased from two dispensaries; both duplicate strain samples were included as a control for retail market consistency. A duplicate sample of one strain (G13) was included as an internal control for panelist consistency. Thus a total of 13 samples was presented to each panelist.

Each stimulus (1 g of dried cannabis flower) was presented in a wide mouth 118 ml (4 oz) amber glass bottle labeled with a three-digit code. Samples were kept in a freezer at -2° C, and thawed at room temperature for two hours before testing. The stimuli were exchanged for fresh samples midway through the study.

In this paper, we identify the odor stimuli according to strain designations provided by the dispensary. The taxonomic validity of named strains is the subject of debate [11] as is the derivation of modern, commercially available, hybrid strains [12]. In their exhaustive treatment of the genus, Clarke and Merlin stated that cannabis presents a “confusing taxonomic situation” and a “challenging botanical problem” [1]. They conclude that “*Cannabis* should be divided into seven taxonomically distinct biotypes of three species with six putative subspecies levels.” In view of these complexities, we note that our use of retail strain designations to identify the samples is purely a matter of convenience. In any case, the precise taxonomic status of our samples is not necessary for our present interest in exploring and characterizing olfactory variation in commercially available offerings.

### Odor descriptors

Forty-eight odor descriptors (Table 1) were culled from online resources aimed at cannabis aficionados, e.g. https://www.leafly.com. Given the exploratory nature of our study, this list was deliberately over-inclusive. The descriptors were printed in alphabetical order on the paper ballot provided with each sample.

**Table 1:** The 48 odor descriptors used to characterize the samples in this study.

ammonia	diesel	mint	skunk
apple	earthy	nutty	spicy
apricot	flowery	orange	strawberry
berry	grape	peach	sweet
blue cheese	grapefruit	pear	tar
blueberry	herbal	pepper	tea
butter	honey	pine	tobacco
cheese	lavender	pineapple	tree fruit
chemical	lemon	plum	tropical fruit
chestnut	lime	pungent	vanilla
citrus	mango	rose	violet
coffee	menthol	sage	woody

### Product evaluation questions

The printed ballot accompanying each stimulus included three product evaluation questions. Each was prefaced with “Based only on the smell” and concluded with either “how potent do you think this sample of cannabis is?”, “how interested would you be in smoking this sample of cannabis?” or “how much would you expect to pay for a gram of this cannabis?” Response options for the first two questions were 11-point scales (with labeled endpoints) ranging from zero (“not potent/not interested at all”) to 10 (“extremely potent/extremely interested”); those for the third question consisted of 11 values ranging in two-dollar increments from $2/g to $22/g.

### Procedure

After signing the consent form, participants were asked whether they had (a) purchased and (b) smoked cannabis flower since it became legal in the state of Colorado on January 1, 2014. Subjects were tested individually in a well-ventilated 6.0 x 2.6 m room with neutral decor. The order of sample presentation was randomized for each subject. Panelists were instructed to sniff the sample *ad libitum* and to check as many or as few odor descriptors as applied to it. It was emphasized that there were no right or wrong answers. The test administrator and all but the current sample bottle were hidden behind an opaque white partition.

### Chemical analysis

All reagents used were of analytic grade. Δ-9-THC (CAS No. 1972-08-3, from Restek Corp., Bellefonte, PA, USA) at a concentration of 0.50 mg/mL in methanol was used as a standard. This solution also contained phenanthracene at a concentration of 0.50 mg/mL which served as an internal standard for chromatography.

Solutions from each sample of cannabis were prepared for chromatographic analysis by harvesting 25 mg of material near the tip of the inflorescence. The material was macerated and suspended in 1.0 ml of methanol containing 0.50 mg/mL of phenanthracene. This suspension was vortexed for 10 min then centrifuged (2,000 g x 10 min). Aliquots of the supernatant were taken for chromatographic analysis. Separate extraction and standard addition experiments quantified the extraction efficiency of this method and demonstrated minimal matrix effects.

Chromatographic analysis was performed on a Hewlett Packard 6890 II GC with a low-polarity capillary column (Rxi-5ms, 30 m, 0.25 mm inner diameter, 0.25 mm thickness, Restek Corp.) followed by a Flame Ionization Detector (FID). Linear flow of the hydrogen carrier gas (99.995% purity) was held at 1.0 mL/min. Samples of volume 0.6 μL were applied to a split injector (1:20) maintained at 250°C. The column oven temperature was programmed as follows: 40°C for 3.0 min then increased to 250°C at a rate of 25°C per min (total time 8.4 min) and finally held at 250°C for 8.6 min (20 min total run time). The detector temperature was maintained at 250°C. The FID signal was digitized (U6-Pro, LabJack Corp., Lakewood, CO, USA) and analyzed (Igor Pro, WaveMetrics, Inc., Portland, OR, USA) using custom built tools.

The reported percentage composition (mass of the individual component per mass of as-received sample) was determined based on relative response in the FID chromatograms. THC identification was based on direct comparison with an authentic sample. All measurements were performed in replicate with a relative standard deviation exceeding 5%.

The chromatograph was tuned daily; analysis of blanks and standards showed no contaminating compounds, and validated instrument response both in signal intensity and retention time.

## Results

### Subject demographics

Sixty-one people (33 men, 28 women; mean age 28.2 ± 8.4 years) took part in the study. Of these, all but three had purchased cannabis since January 1, 2014, and all but one subject had smoked it. The high rates of purchase (95.1%) and use (98.4%) among study participants occurred despite efforts to recruit former and non-users as well. As an unintended result, the study resembles a typical consumer perception study where purchase, use, and/or familiarity with the product is a requirement of participation.

### Descriptor usage

Participants rated the 13 samples using the check-all-that-apply list of 48 descriptors (Table 1). The number of descriptors endorsed per sample by a given participant (mean across 13 samples) ranged from 1.9 to 11.8; i.e., some participants used descriptors more sparingly than others. The overall mean (± SD) number of descriptors per participant was 5.2 ± 2.8 (*n* = 61).

**Fig 1:**
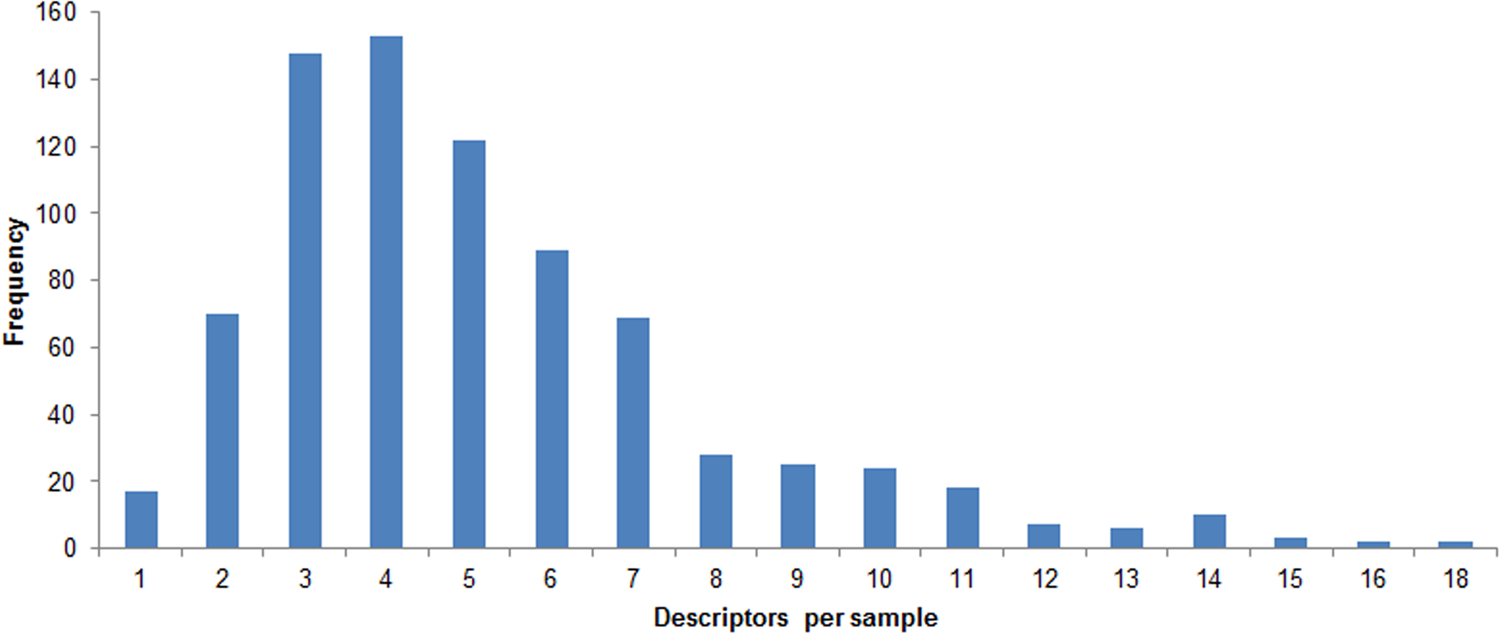
Frequency distribution of number of descriptors applied to each sample by each participant. Maximum number of descriptors for a given sample was 48. The total number of sample evaluations displayed is 793.

The mean number of odor descriptors assigned to a cannabis sample ranged from 4.5 (Lemon Diesel) to 6.1 (Jilly Bean) (overall mean = 5.2). The number of odor descriptors endorsed for a given sample by a given participant ranged from 1 to 18 (Fig 1). Endorsement frequencies of 3, 4, and 5 accounted for 423 or 53.3% of all sample evaluations. Endorsement frequency ≥10 accounted for 71 or only 9% of all sample evaluations. These results raise the possibility that the strains examined here might be adequately described with fewer than 48 odor descriptors.

**Fig 2:**
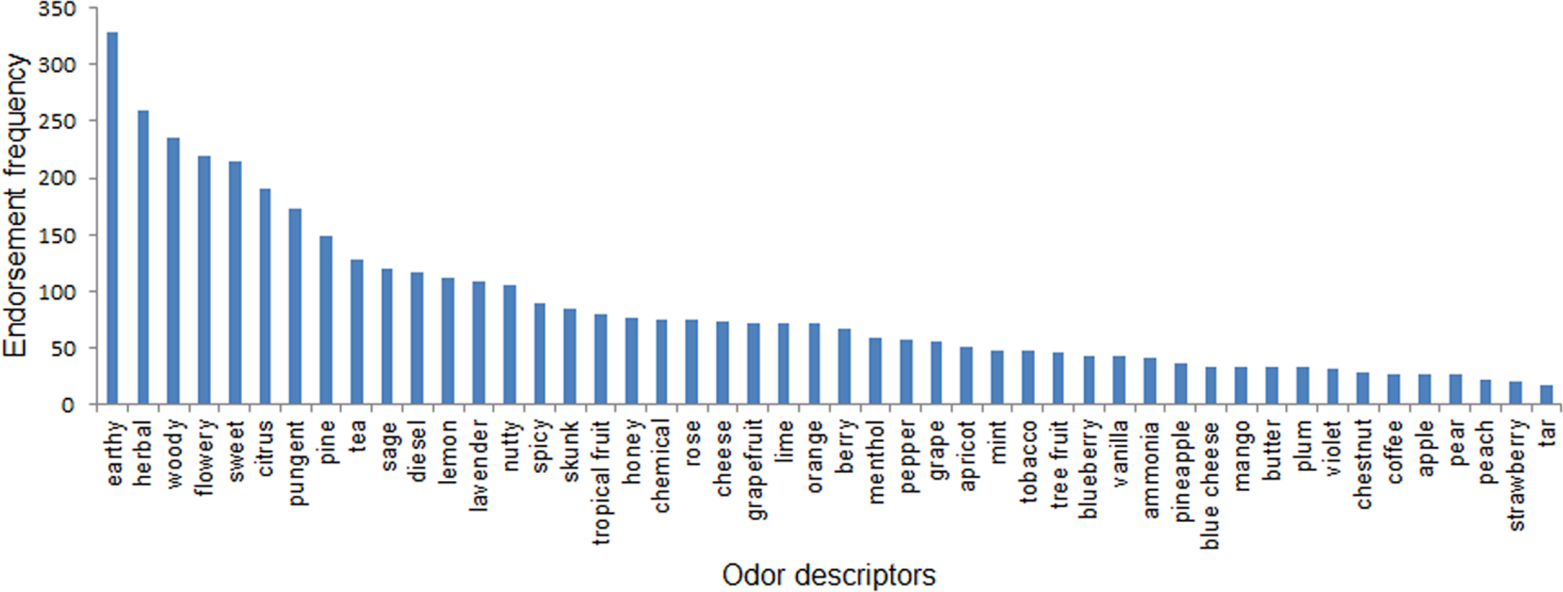
Endorsement frequencies summed across all 13 samples and 61 test subjects.

Frequency of use for each of the 48 descriptors is shown in Fig 2. The most frequently used descriptor was *earthy* (328 endorsements or 7.9% of total); the least frequently used descriptor was *tar* (17 endorsements or 0.4% of total). The 11 most frequently used descriptors accounted for 51.5% of all endorsements, while the top 23 accounted for 76.2% and the top 34 for 90.2%. That the 15 least frequently used descriptors accounted for only 10.8% of total endorsements indicates that many odor descriptors were only rarely used. The most frequently used descriptors were from familiar categories of everyday experience such as woods, spices, florals, and citrus fruits, but also included notes such as *diesel*, *skunk*, *pungent* and *cheese*.

### Strain-specific aroma profiles

**Fig 3:**
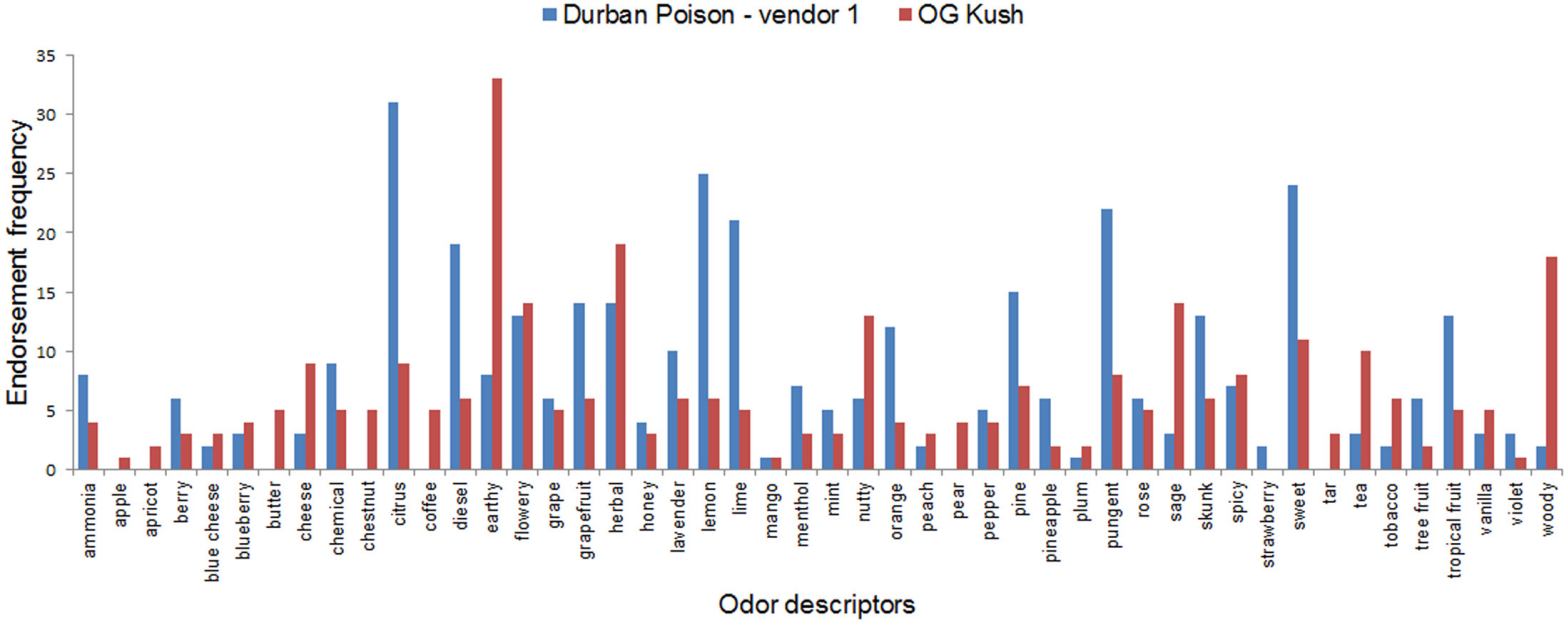
Aroma profiles for two of the strains tested in this study. Frequency counts of each odor descriptor.

A strain’s aroma profile can be represented as frequency counts across the odor descriptors. Two examples are provided in Fig 3. Visual inspection reveals striking differences between OG Kush and Durban Poison (vendor 1). The most common terms used to describe OG Kush are *earthy*, *herbal*, and *woody*, along with *flowery*, *sage*, and *nutty*. In contrast, Durban Poison (vendor 1) was characterized as *citrus*, *lemon*, and *sweet*, as well as *pungent*, *lime*, and *diesel*.

### Hierarchical cluster analysis

**Fig 4:**
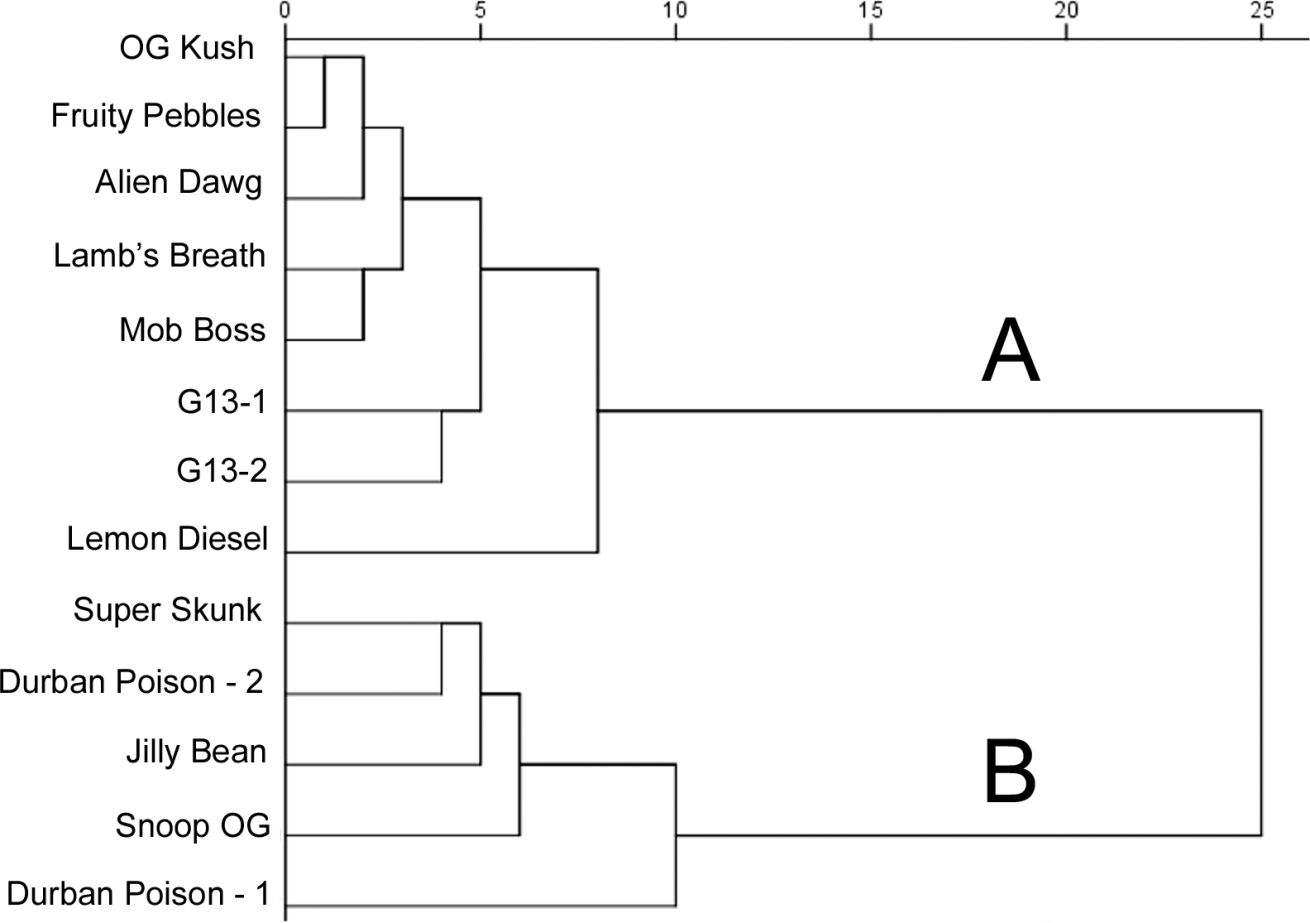
Hierarchical clustering analysis of all 13 cannabis samples. Distances are rescaled to 25. Samples in Cluster A were characterized as *earthy*, *woody*, and *herbal*; those in Cluster B as *citrus*, *lemon*, *sweet*, and *pungent* (see text).

Using endorsement frequencies of the 48 descriptors from all 61 participants as input data, the cannabis samples were analyzed with the Hierarchical Cluster procedure in IBM SPSS Version 23, using between-groups linkage on squared Euclidean distances. The resulting dendrogram (Fig 4) suggested two clusters, with Cluster A consisting of eight stimuli and Cluster B of five. Cluster A includes both duplicate samples of strain G13, indicating that participants used the odor descriptors consistently across samples. Cluster B includes both independent retail samples of Durban Poison, indicating olfactory consistency in retail offerings (at least for this strain).

By referencing the odor descriptor frequencies for each sample it is possible to characterize the olfactory profiles of each cluster. The most frequent descriptors for samples in Cluster A were uniformly *earthy*, *woody*, and *herbal*. For samples in Cluster B, the most frequent descriptors included *citrus*, *lemon*, *sweet*, and *pungent*.

Each cluster had one less closely associated sample (Lemon Diesel in Cluster A; Durban Poison (vendor 1) in Cluster B). Research with more refined measures (e.g., multipoint rating scales) and a larger number of strains may provide evidence that these samples constitute distinct clusters of their own. Under the present circumstances, however, the most parsimonious interpretation is that they segregate with the two primary clusters.

Of potential concern is the large number of clustering variables relative to the number of stimuli [13]. This raises the possibility that inter-correlation between variables could lead to common odor notes being overrepresented in the cluster solution. Principal Components Analysis or a similar technique is sometimes used to reduce the number of clustering variables. A known risk of this approach, however, is the potential loss of valuable information [13]. This risk is particularly acute in this study, which is an initial attempt to identify relevant items for a cannabis sensory lexicon. Therefore, we took two different approaches to assess the robustness of the cluster solution.

Because the odor descriptors used as clustering variables included the term “citrus” as well as four specific citrus items (grapefruit, lemon, lime, orange), it is possible that shared aspects of these variables were overrepresented in the clustering solution. To address this possibility, we ran another HCA in which the descriptors grapefruit, lemon, lime, and orange were omitted from the analysis. The resulting dendrogram suggested two clusters consisting of the same strains as in Fig 4, and with nearly identical within-cluster arrangements. This suggests that the basic division of the stimuli into two clusters is not driven by an overrepresentation of citrus-related terms in the odor descriptor set.

Next, to assess the robustness of the cluster solution using fewer clustering variables, we re-ran the cluster analysis but omitted the 15 least frequently endorsed items. While differing in some details, the resulting dendrogram produced two clusters comprised of the same stimuli as in Fig 4. Subsequent HCA runs that omitted increasing numbers (20, 25, 30, and 35) of the least frequently endorsed descriptors produced similar results. It thus appears that cannabis flowers of various strains cluster robustly based on olfactory profiles consisting of a handful of descriptive terms (e.g., *earthy*, *herbal*, *woody*, *flowery*, *sweet*, *citrus*, *pungent*, and *pine*). Another implication is that a useful cannabis lexicon may be obtained with fewer than 48 odor descriptors.

**Fig 5:**
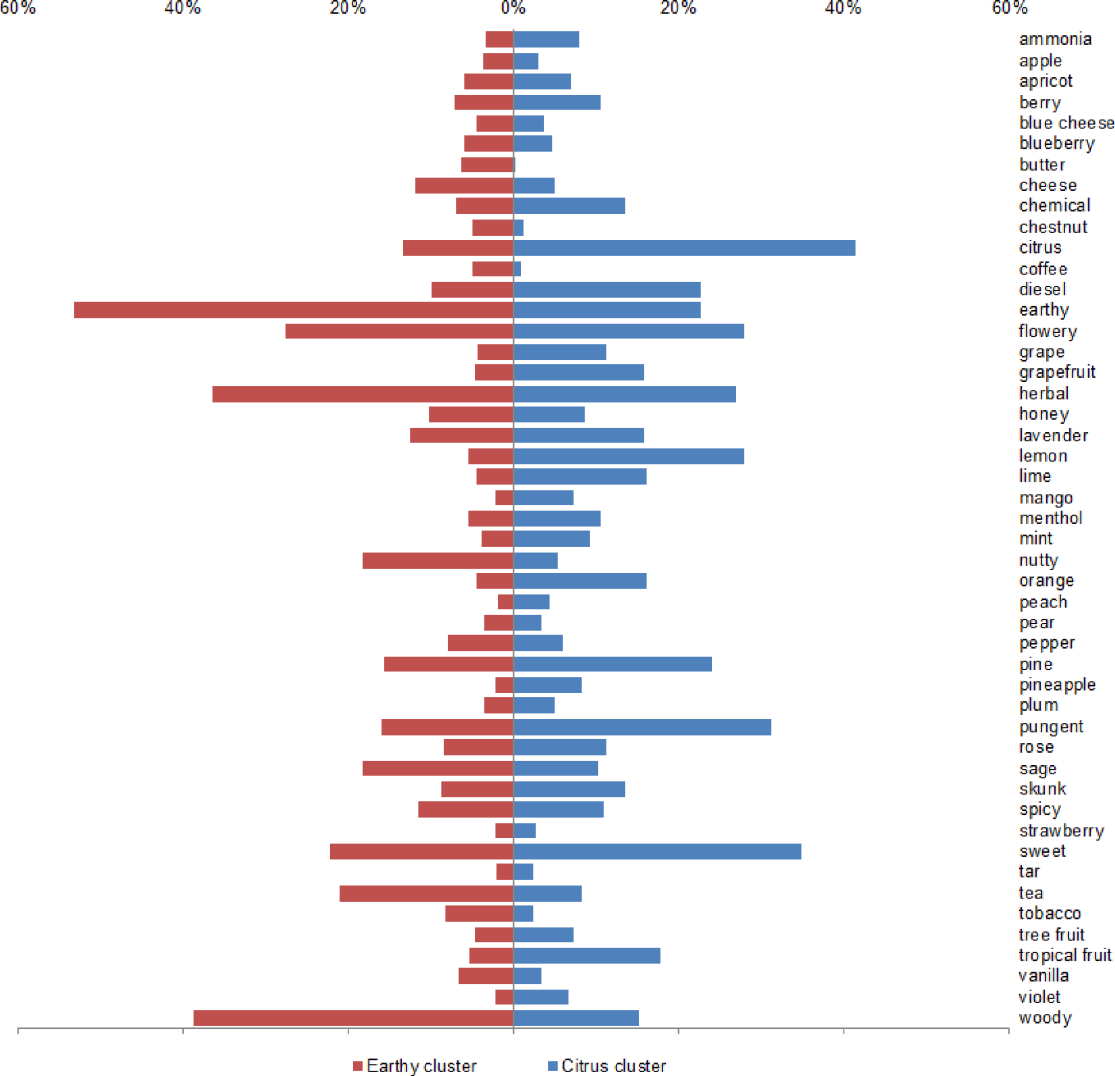
**Composite aroma profiles for samples in Cluster A (Earthy) and Cluster B (Citrus).** Percentages refer proportion of observations in which the odor descriptor was endorsed. There were 5 samples in the Citrus cluster and 8 samples in the Earthy cluster.

To further characterize the clusters identified in Fig 4, a composite odor profile was created for each of them (Fig 5). It displays descriptor endorsements as a percentage of the possible opportunities (samples x participants) for the samples in Cluster A (*n* = 488) and Cluster B (*n* = 305). Some descriptors (e.g., *spicy*) were endorsed in roughly equal proportions in both clusters. Others (e.g., *earthy*) are more heavily represented in one cluster than the other. The composite aroma profiles are consistent with the characterization of Cluster A as *earthy*, *herbal*, and *woody*, and Cluster B as *citrus*, *lemon*, *sweet*, and *pungent*.

### Product evaluation questions

For each sample, means were calculated for smell-based estimates of potency (Potency), interest in smoking (Interest), and estimated cost per gram (Price) (Table 2). The strain means varied by about 2 rating points on Potency and Interest, and by more than $3.00 on Price. The smell-based estimated prices corresponded reasonably well to the actual purchase price per gram, which ranged from $9.29 to $12.24.

**Table 2:** Cluster membership and smell-based product evaluation ratings for each sample.

Sample	Cluster	Potency	Interest	Price ($)
Durban Poison vendor 1	Citrus	8.3 ± 1.5	8.8 ± 1.4	14.00 ± 4.08
Durban Poison vendor 2	Citrus	8.1 ± 1.6	8.2 ± 2.2	13.61 ± 3.67
Super Skunk	Citrus	7.8 ± 1.4	8.3 ± 1.6	13.21 ± 3.71
Jilly Bean	Citrus	7.7 ± 1.6	8.3 ± 1.9	13.15 ± 3.84
Snoop OG	Citrus	7.2 ± 1.8	7.6 ± 1.9	11.71 ± 3.38
Lamb’s Breath	Earthy	7.2 ± 1.6	7.4 ± 1.9	12.20 ± 3.96
Mob Boss	Earthy	7.1 ± 1.6	7.3 ± 2.2	11.80 ± 3.72
Fruity Pebbles	Earthy	6.7 ± 1.8	7.1 ± 2.2	11.57 ± 3.52
G13–2	Earthy	6.7 ± 1.7	7.1 ± 2.2	11.64 ± 4.11
Alien Dawg	Earthy	6.5 ± 1.6	7.0 ± 2.0	11.64 ± 3.91
Lemon Diesel	Earthy	6.4 ± 1.8	6.4 ± 2.5	10.66 ± 3.86
G13–1	Earthy	6.3 ± 1.9	6.7 ± 2.4	10.98 ± 4.06
OG Kush	Earthy	6.3 ± 1.9	6.8 ± 2.2	10.89 ± 3.38

Ratings are given as mean ± SD. Cluster membership as in Fig 4. Samples are identified by strain names supplied by the retail dispensary; this does not imply definitive taxonomic status (see text).

To test whether differences between samples were not spurious, we conducted a repeated-measures ANOVA on each product quality variable. (The sample size did not provide sufficient residual degrees of freedom for us to use the preferred statistical approach, namely a doubly multivariate design with Sample (13) and Product Quality (3) as the repeated-measures factors [14].) Because Mauchly’s test was significant (*p* < 0.001), sphericity could not be assumed and therefore conservative tests of within-subjects effects were required. Accordingly, we present only Greenhouse-Geisser values. All three variables varied significantly across odor samples: Potency *F*(9.2, 548.2) = 14.99, *p* < 0.001; Interest *F*(8.3, 499.3) = 12.28, *p* < 0.001; and Price *F*(8.7, 520.7) = 13.26, *p* < 0.001. We conclude that some strains were perceived as higher/lower than others on Potency, as well as on Interest and Price.

### Product evaluations and aroma

The cluster membership of each sample (based on the HCA results in Fig 4) is provided in Table 2. It appears that samples in the Citrus group (Cluster B) were perceived as more potent than those in the Earthy group (Cluster A), and also yielded higher scores for smoke interest and estimated price. To test this observation statistically, a single planned comparison of Cluster A (Earthy) versus Cluster B (Citrus) was carried out using aggregated ratings for each variable in a repeated measures ANOVA. The results confirm a statistically significant difference between sample clusters for each product evaluation variable: Potency *F*(1, 60) = 88.01, *p* < 0.001; Interest *F*(1, 60) = 86.58 *p* < 0.001; Price *F*(1, 60) = 98.23, *p* < 0.001. Group means (± SD) were 7.8 ± 1.1 vs 6.6 ± 1.2 for Potency; 8.2 ± 1.2 vs 6.9 ± 1.5 for Interest; and 13.13 ± 3.07 vs 11.42 ± 3.18 for Price (Cluster B/Citrus versus Cluster A/Earthy, respectively). These results indicate that group-level differences in strain aroma are linked to significant differences in consumer estimates of product quality. In particular, the *citrus*, *lemon*, *sweet*, and *pungent* character of Cluster B is associated with greater perceived quality than the *earthy*, *herbal*, and *woody* character of Cluster A.

### THC concentration

The THC concentration of each sample as determined by our chemical analysis is listed in Table 3. There was a 3.5-fold difference in concentration between the sample with the least THC and the sample with the most. Colorado regulations require that THC concentrations be stated on the retail label as a range representing the lowest and highest percentage values drawn from every test conducted on a given strain, from the same cultivation facility, within the last six months. Our experimentally determined THC content was lower than the range of values listed on the retail label for at least 6 of the 12 samples (see S2 Note. THC experimental versus label). A similar overstatement of THC content has been reported for edible cannabis products from California and Washington [15].

**Table 3:** Measured THC content of the cannabis samples used in this study.

Sample	THC content (%)
Durban Poison–vendor 2	27.1
Lemon Diesel	22.7
OG Kush	21.9
Fruity Pebbles	21.5
Snoop OG	19.1
Jilly Bean	17.4
Durban Poison–vendor 1	15.1
Mob Boss	11.7
Alien Dawg	11.2
Super Skunk	10.4
G13	7.9
Lamb’s Breath	7.7

Samples are identified by strain names supplied by the retail dispensary; this does not imply definitive taxonomic status (see text).

To determine whether perceived potency based on smell varied systematically with actual THC concentration, we calculated the Spearman rank-order coefficient between THC value and mean Potency rating for each strain. Because samples G13-1 and G13-2 were split samples of the same physical material, we measured THC in strain G13 only once. Rather than arbitrarily pair the THC data with the mean Potency of one sample or the other, we ran the analysis twice. We calculated the Spearman correlation coefficient once using the value for G13-1, and again using the value for G13-2. In neither case was perceived potency correlated with measured THC (using G13-1, r_s_ = -.021, *ns*; using G13-2, r_s_ = -.098, *ns*; n = 12 in each analysis). Thus, while participants perceived large differences in sample potency based on smell, these perceptions do not correspond to the THC content of the samples. Similar analyses showed that neither Interest nor Price were correlated with THC content (S3 Note. Experimental THC content versus Interest and Price).

In addition, correlation analysis of the Product Evaluation variables versus both the lower- and upper-range THC values reported on the retail labels also found no evidence of a relationship (see S4 Note. On-label THC content versus product evaluations).

## Discussion

In order to create an empirically validated olfactory lexicon for the aroma of dried cannabis flower, participants were asked to characterize sniff samples using a check-all-that-apply descriptor ballot that included 48 cannabis-relevant odor terms drawn from a variety of informed sources. The sniff samples consisted of 11 strains selected to span the olfactory gamut and purchased at local dispensaries. While untrained in sensory description, study participants were nearly all smokers and purchasers of legally available cannabis, and were therefore familiar with the product.

We found that untrained panelists are able to discriminate bud aroma from different cannabis strains. Duplicate samples of G13 were rated similarly, as were two samples of Durban Poison purchased from different vendors. Thus, not only can consumers discriminate the different strains of cannabis, they can do so consistently. This comports with the ability of untrained odor judges to discriminate among varieties of fresh hops—a natural product closely related to cannabis [16].

The aroma differences among strains are substantial. Although we began this exploratory study with no a priori hypothesis regarding olfactory relationships, hierarchical cluster analysis revealed two distinct groups: Cluster A consisting of strains characterized as *earthy*/*woody*/*herbal*, and Cluster B containing strains perceived as *citrus*/*lemon*/*sweet*/*pungent*. Given their distinctive aroma profiles, and the large statistical distance between them, we propose that these clusters represent a high-level divide in the cannabis bud odor space. This proposition can be tested in the future by sensory evaluation of additional samples drawn from the approximately 600 named varieties of cannabis [12]. It is tempting to compare the groupings described here to the much remarked-upon odor difference between *sativa* and *indica* types [17]. *C*. *sativa* L. is said to have a “spicy or sweet aroma” [1] while *C*. *indica* ssp. *afghanica* and its many hybrids are described as “acrid-smelling” [1, 18]. Determining the precise taxonomic identity of our retail samples is beyond the scope of this study. Our methods, however, have the potential to clarify informal observations regarding the smell of *sativa* and *indica*, and may also reveal more nuanced olfactory phenotypes among individual strains and hybrids.

Whether lower-level relationships within the two main clusters are consistent, and how they might be characterized in olfactory terms, are questions for future research. The use of Likert interval scales rather than dichotomous checklists could bring precision to the classification process. It is clear, however, that statistical classification techniques can illuminate the aromatic relationships among strains. Similar techniques have been used to map strains according to cannabinoid and terpene content [19, 20, 21] as well as microsatellite genetic loci [22].

Our fundamental objective is to provide consumers and experts with an easily understandable set of standardized descriptive terms. Our set of 48 descriptors appears sufficient to cover the relevant odor space and allow consumers to differentiate strains by smell. The size of this set is roughly in keeping with the 85 descriptors used for coffee [8] and the 86 used for wine [7]. Nevertheless, our results also suggest that a smaller subset of odor terms may prove adequate for practical applications.

Most of the descriptors used in this study are self-explanatory and easily defined. A few, such as *diesel*, require further definition and the development of odor standards. A peculiarity of cannabis is that some descriptors with nominally negative connotations (*skunk*, *pungent*, *diesel*) were associated with positive product evaluations, while others (*earthy*, *chemical*, *tar*) were linked with negative evaluations. This is in contrast to wine and coffee terminology in which *skunk*, for example, denotes only a product defect. That disagreeable smelling cannabis strains are popular with certain consumers is well known [23] and resembles the popularity of other stinky products such as cheese, fermented soy, etc. [24].

Another goal of this study was to determine the effect of strain-specific aroma on consumer perceptions of product quality. Participants were asked to rate, based on smell alone, the potency of the sample, how interested they were in smoking it, and how much they would expect to pay for it. Ratings for all three variables differed significantly across samples, indicating that a strain’s scent can impact how consumers rate these aspects of product quality. We found that strains in the *citrus*/*lemon*/*sweet*/*pungent* cluster generated higher estimates of potency, interest, and price than those in the *woody*/*earthy*/*herbal* cluster. Although smell-based consumer perceptions of cannabis potency vary by strain, they show no relationship to experimentally measured THC content. This is not surprising given that THC is nonvolatile and odorless [25]. Nonetheless, the fact that specific odor notes are associated with impressions of product quality could have important implications for the branding and marketing of recreational cannabis.

We note that nearly all study participants had experience in the purchase and use of cannabis, and thus were perhaps more likely to be familiar with descriptive terms unique to the product (e.g., *diesel*). This potential bias did not extend to familiarity with the use of sensory evaluation techniques to describe cannabis. One positive result of this self-selection bias is that price and potency results are more compelling than had they been obtained from cannabis-naive individuals. The fact that participants familiar with cannabis flower and its psychoactive effects believed strain aroma to be related to potency, despite its lack of correlation with THC content, speaks to the power of consumer preconceptions.

What is the physical basis of the odor differences observed here? As many as 140 terpenes have been identified in cannabis of which 17 are found frequently [26]. It is widely held that terpenes are the basis for cannabis bud aroma [27, 28], an assumption based largely on their relative abundance in the plant. Few studies have attempted to relate odor character directly to chemical composition. Mediavilla and Steinemann [29] had volunteers rate the steam-distilled essential oils of *Cannabis* flowers (hemp and drug cultivars) on a 5-point scale from “very bad” to “very good.” They noted an association between high sesquiterpene concentration and low ratings on the one hand, and high monoterpene concentration and high ratings on the other. Rice and Koziel [30] applied psychophysical reasoning to cannabis volatiles collected by SPME from samples in a police evidence room. They highlighted compounds present at a concentration greater than their odor detection threshold, i.e., those most likely to contribute to the perceptible smell. Most were terpenes. It is not obvious how the mono- and sesquiterpenes in cannabis can be responsible for the *skunk*, *diesel*, and *earthy* notes observed in our samples. These characteristic notes may result from synergistic effects among terpenes or from the overlooked presence of non-terpene compounds.

Sensory evaluation, combined with chemical analysis, has the potential to identify the key aroma compounds in cannabis and how they vary across strains. Understanding these relationships will yield practical applications in standards for quality control and product consistency, and will facilitate product development, innovation, and consumer education.

## Supporting information

S1 Note Source and specification of study materials. (DOCX)

S2 Note THC experimental versus label. (DOCX)

S3 Note Experimental THC content versus Interest and Price. (DOCX)

S4 Note On-label THC content versus product evaluations. (DOCX)

S1 Table Raw sensory ratings and participant demographics. (XLSX)
