# Supplementary material for: Consumer perceptions of strain differences in Cannabis aroma
Source: PLoS One. 2018 Feb 5;13(2):e0192247. doi: 10.1371/journal.pone.0192247 (PMC5798829; doi:10.1371/journal.pone.0192247)
Supplement: S1 Note — (DOCX) [file pone.0192247.s001.docx]

**S1 Note. Source and specification of study materials**

In this note we provide details of the commercial sources and on-label specifications of the cannabis used as odor stimuli.

Dispensary #1

Solace Meds, LLC

301 Smokey Street, Unit A

Fort Collins, CO 80525

Retail license: 402R-00239

Information provided on the printed retail labels from Solace Meds, LLC:

Strain: Super Skunk

Strain description: “Sativa Dominant Hybrid”

Cultivation facility RMCF license: 403R-00538

Harvest date: 1/16/2017

THC content: 0.00-16.96% THC-A

CBD content: 0.00-0.00% CBD-A

CBG content: 0.00-0.83% CBG-A

Strain: Lemon Diesel

Strain description: “50/50 Hybrid”

Cultivation facility RMCF license: 403R-00317

Batch number: RW021517B2P1 LD

THC content: 24.54-25.71% THC-A

CBD content: 0.00-0.00% CBD-A

Strain: Durban Poison (designated “vendor 1” sample in text of paper)

Strain description: “Sativa Dominant Hybrid”

Cultivation facility RMCF license: 403R-00538

Batch number: RW021317B1P4 DP

THC content: 17.29-19.54% THC-A

CBD content: 0.00-0.00% CDB-A

CBN content: 0.00-0.37% CBN

Strain: Fruity Pebbles

Strain description: “50/50 Hybrid”

Cultivation facility RMCF license: 403R-00317

Batch number: RW021517B2P1 FP

THC content: 17.28-25.79% THC-A

CBD content: 0.00-0.00% CDB-A

CBG content: 0.00-0.53% CBG-A

Strain: OG Kush

Strain description: “Indica”

Cultivation facility RMCF license: 403R-00317

Batch number: RW021317B1P4 OGK

THC content: 0.00-23.61% THC-A

CBD content: 0.00-0.00% CDB-A

Dispensary #2

Green Meadows Wellness LLC

d/b/a Infinite Wellness Center

900 North College Avenue

Fort Collins, CO 80524

Retail license: 402R-00235

Information provided on the printed retail labels from Infinite Wellness Center:

Strain: Durban Poison (designated “vendor 2” sample in text of paper)

Strain description: [none provided]

Cultivation facility RMCF license: 403R-00620

Harvest date: 1/16/2017

THC content: 20.7-29.57%

Strain: Alien Dawg

Strain description: [none provided]

Cultivation facility RMCF license: 403R-00311

Harvest date: 2/13/2017

THC content: 26.5-29.75%

Strain: Mob Boss

Strain description: [none provided]

Cultivation facility RMCF license: 403R-00311

Harvest date: 1/9/2017

THC content: 21.9-25.7%

Strain: G13

Strain description: [none provided]

Cultivation facility RMCF license: 403R-00620

Harvest date: 1/30/2017

THC content: 23.5-29.3%

Strain: Snoop OG

Strain description: [none provided]

Cultivation facility RMCF license: 403R-00620

Harvest date: 1/23/2017

THC content: 28.7-33.69%

Strain: Lamb’s Breath

Strain description: [none provided]

Cultivation facility RMCF license: 403R-00620

Harvest date: [none provided]

THC content: 24.0-31.4%

Strain: Jilly Bean

Strain description: [none provided]

Cultivation facility RMCF license: 403R-00311

Harvest date: 11/28/2016

THC content: 22.0-33.0%

Retail Marijuana Cultivation Facility license holders

RMCF: 403R-00311

Green Meadows Wellness LLC

d/b/a Infinite Wellness Center

900 North College Avenue

Fort Collins, CO 80524

RMCF: 403R-00317

Solace Meds LLC

1724-1732 Willox Court

Fort Collins, CO 80524

RMCF: 403R-00538

Keens Greens Edibles LLC

1015 West Evans Avenue, Unit EB

Denver, CO 80223

RMCF: 403R-00620

Green Meadows Wellness LLC

d/b/a Infinite Wellness Center

1717 Willox Court, Units A and B

Fort Collins, CO 80524
