# Supplementary material for: Consumer perceptions of strain differences in Cannabis aroma
Source: PLoS One. 2018 Feb 5;13(2):e0192247. doi: 10.1371/journal.pone.0192247 (PMC5798829; doi:10.1371/journal.pone.0192247)
Supplement: S2 Note — (DOCX) [file pone.0192247.s002.docx]

**S2 Note. THC experimental versus label**

The THC concentration of each sample as determined by our chemical analysis (taken from Table 3 in the main paper) is listed in Table S3 below. For purposes of comparison, we also list the THC concentrations printed on the retail labels provided by the dispensaries (taken from S1 Note. Source and specification of study materials).

The data in Table A reveal discrepancies between labeled and actual potencies. In only 4 of 12 samples did the measured THC concentration fall within the range stated on the retail label; in the remaining 8 samples the measured concentration was below the minimum labeled range.

According to Colorado marijuana regulations, THC tests have an allowable plus or minus 15% variance. Two of the 8 out-of-range samples (Jilly Bean and Durban Poison – vendor 1) move into range when this allowable variation is taken into account. But even then, only 6 of 12 samples contained as much THC as labeled.

The discrepancies were not localized to a particular retailer or cultivation facility. We do not know the identity of the licensed testing lab (or labs) that determined the THC values reported on the retail labels. For the cannabis samples studied here, however, it appears that the retail labels tend to overstate the potency of the product.

Table S2. Measured vs labeled THC content (%) of cannabis samples.

Experimental THC values are given to one decimal place; label THC values are provided exactly as printed on the retail label.
