# Supplementary material for: Consumer perceptions of strain differences in Cannabis aroma
Source: PLoS One. 2018 Feb 5;13(2):e0192247. doi: 10.1371/journal.pone.0192247 (PMC5798829; doi:10.1371/journal.pone.0192247)
Supplement: S3 Note — (DOCX) [file pone.0192247.s003.docx]

**S3 Note. Experimental THC content versus Interest and Price**

The Spearman correlation coefficient (n = 12) between experimentally measured THC concentration and product evaluation variable Interest was *r*_s_ = -.105, *p* = n.s., using the mean Interest rating for sample G13-1; it was *r*_s_ = -.175, *p* = n.s. using the mean Interest rating for sample G13-2.

For the same analysis between THC concentration and the product evaluation variable Price, the result was *r*_s_ = -.161, *p* = n.s. using the mean Price rating for sample G13-1; it was *r*_s_ = -.217, *p* = n.s. for sample G13-2.

Regardless of which sample’s data was used for the duplicated strain G13, the results were the same: there was no correlation between THC content and either product evaluation variable (Interest or Price). A lack of correlation between THC content and the third product evaluation variable (Potency) was reported in the main text of the paper.
