# Supplementary material for: Consumer perceptions of strain differences in Cannabis aroma
Source: PLoS One. 2018 Feb 5;13(2):e0192247. doi: 10.1371/journal.pone.0192247 (PMC5798829; doi:10.1371/journal.pone.0192247)
Supplement: S4 Note — (DOCX) [file pone.0192247.s004.docx]

**S4 Note. On-label THC content versus product evaluations**

We report here the Spearman correlation coefficients (r_s_) between the product evaluation variables (Potency, Interest, and Price) and each of the on-label values of THC content (Lower Value, Upper Value). In parallel with the analyses run on measured THC (see main text and S4 Note. Experimental THC content versus Interest and Price variables), each correlation analysis (n = 12) was run twice: once with the data from sample G13-1, and again with the data from sample G13-2.

Table S4. Spearman correlations coefficients (r_s_) for product evaluation variables and on-label THC values. None of the comparisons approached statistical significance, i.e., all p >> 0.10.
